# Algorithm for the reconstruction of the parotid region: a single institution experience

**DOI:** 10.1186/s12903-024-03872-z

**Published:** 2024-01-18

**Authors:** Chun-Bo Dou, Si-Rui Ma, Shi-Long Zhang, Heng Su, Zi-Li Yu, Jun Jia

**Affiliations:** 1 Department of Oral and Maxillofacial Surgery, School and Hospital of Stomatology, Wuhan University, Wuhan, 430079 China; 2 Dongfeng Stomatological Hospital, Hubei University of Medicine, Shiyan, China; 3 State Key Laboratory of Oral & Maxillofacial Reconstruction and Regeneration, Key Laboratory of Oral Biomedicine Ministry of Education, Hubei Key Laboratory of Stomatology, School & Hospital of Stomatology, Wuhan University, Wuhan, China

**Keywords:** Parotid malignant tumors, Soft tissue flap, Free flaps, immediate reconstruction

## Abstract

**Objective:**

This study aims to discuss the characteristics and treatment methods of malignant tumors in the parotid region, as well as the therapeutic effects of immediate free flap reconstruction of soft tissue for postoperative defects.

**Materials and methods:**

A retrospective review was conducted on 11 cases of soft tissue flap reconstruction for postoperative defects following the resection of malignant tumors in the parotid region. Statistical analysis was performed based on clinical data.

**Results:**

Among the 11 cases of malignant tumors in the parotid region, there were 2 cases of secretory carcinoma (SC) of the salivary gland, 2 cases of squamous cell carcinoma (SCC), 2 cases of carcinosarcoma, 1 case of mucoepidermoid carcinoma (MEC), 1 case of epithelial-myoepithelial carcinoma (EMC), 1 case of salivary duct carcinoma (SDC), 1 case of basal cell carcinoma (BCC), and 1 case of osteosarcoma. Among these cases, 4 were initial diagnoses and 7 were recurrent tumors. The defect repairs involved: 8 cases with anterolateral thigh free flap (ALTF), 2 cases with pectoralis major muscle flaps, and 1 case with forearm flap. The size of the flaps ranged from approximately 1 cm × 3 cm to 7 cm × 15 cm. The recipient vessels included: 4 cases with the facial artery, 4 cases with the superior thyroid artery, and 1 case with the external carotid artery. The ratio of recipient vein anastomosis was: 57% for branches of the internal jugular vein, 29% for the facial vein, and 14% for the external jugular vein. Among the 8 cases that underwent neck lymph node dissection, one case showed lymph node metastasis on pathological examination. In the initial diagnosis cases, 2 cases received postoperative radiotherapy, and 1 case received ^125^I seed implantation therapeutic treatment after experiencing two recurrences. Postoperative follow-up revealed that 2 cases underwent reoperation due to local tumor recurrence, and there were 2 cases lost to follow-up. The survival outcomes after treatment included: one case of distant metastasis and one case of death from non-cancerous diseases.

**Conclusion:**

Immediate soft tissue flap reconstruction is an important and valuable option to address postoperative defects in patients afflicted with malignant tumors in the parotid region.

## Introduction

Parotid malignant tumors encompass both primary tumors originating in the parotid gland and metastatic tumors, comprising approximately 20 to 35% of all parotid tumors [1–4]. In our clinical experience, we have observed limited cases of free flap reconstruction following resection of parotid malignant tumors. This could be attributed to the fact that more than 80% of parotid tumors are located in the superficial lobe of the gland, allowing for early detection (clinical stage I or II). Additionally, most tumors have minimal adhesion to the skin, enabling preservation and closure of the wound [5]. In cases of small to moderate defects involving only a portion of the skin, adjacent pedicled flaps can yield satisfactory outcomes [6, 7]. However, we often encounter stage III or IV malignant tumors or recurrent tumors, where tumor resection results in challenging closure of skin and deep tissue defects. In such situations, neighboring tissue flaps may not provide sufficient volume of subcutaneous tissue and can lead to postoperative depression deformity. Therefore, reconstructions using free soft tissue flaps such as anterolateral thigh flaps, forearm flaps, or pectoralis major muscle flaps become necessary.

Due to the limited number of clinical cases and literature reports concerning these situations, we have gathered 11 cases of immediate soft tissue reconstruction after extensive resection of parotid region malignant tumors treated at the Department of Oral and Maxillofacial Surgery, School and Hospital of Stomatology, Wuhan University, from October 2012 to June 2023. In this report, we present their clinical diagnosis and treatment to serve as a reference for clinicians encountering similar cases in their clinical practice.

## Methods and patients

**Fig. 1:**
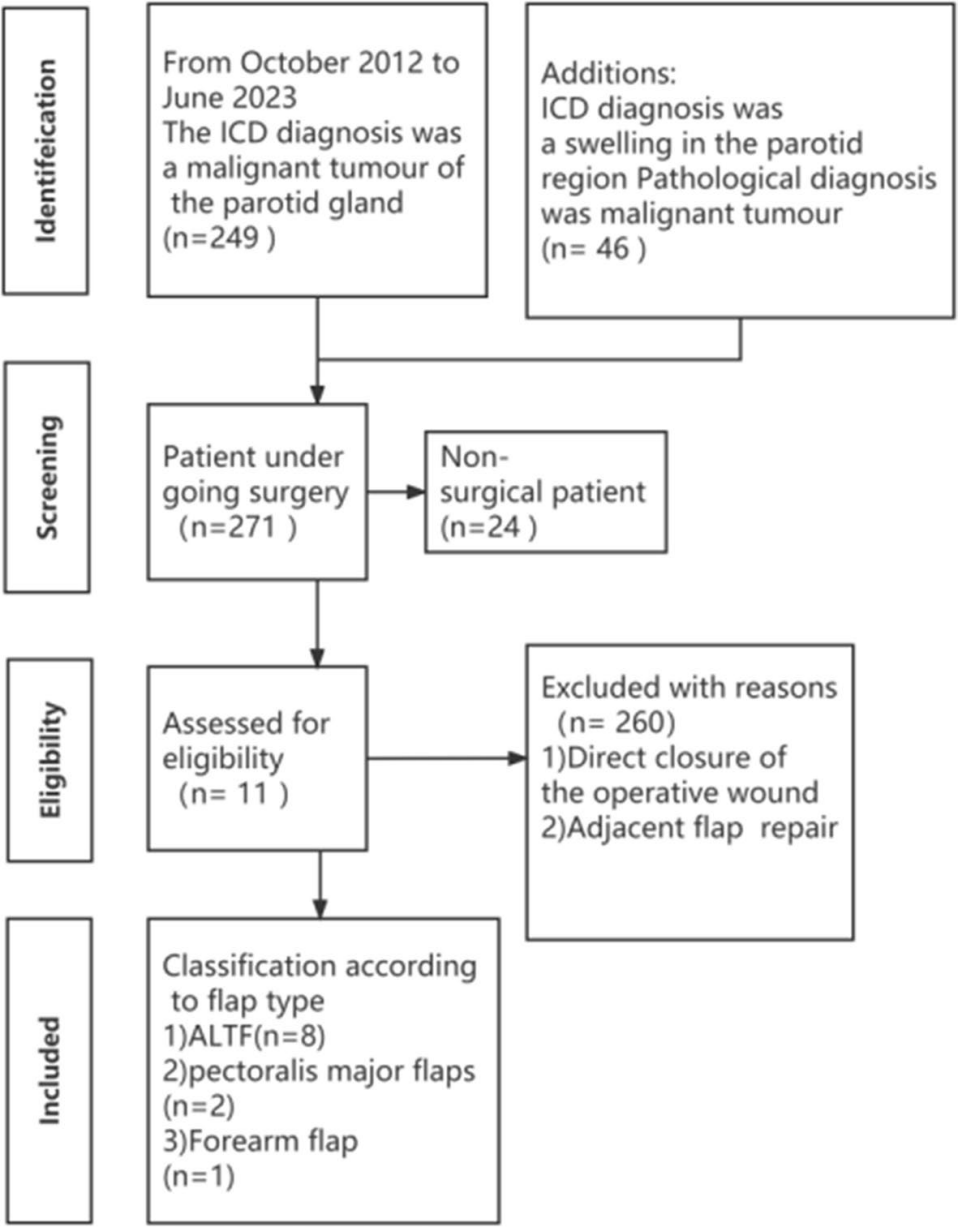
Flow diagram of case search and inclusion and exclusion criteria

This retrospective study was approved by the Review Board of the Ethics Committee of the School and Hospital of Stomatology, Wuhan University. The flow diagram illustrating the case search and selection criteria is depicted in Fig. 1. The collected data consisted of demographic information, pathological diagnosis, microsurgery characteristics, and preoperative, intraoperative and postoperative photos. Additionally, postoperative complications, survival outcomes and their corresponding management were recorded and analyzed.

## Results

### Patient summary

A retrospective review was conducted on data from 11 patients. The characteristics of these patients were summarized in Table 1**.** The statistical analysis of the characteristics of the 11 patients were shown in Table 2. Among the 11 cases of malignant tumors in the parotid region, there were 6 males and 5 females (male-to-female ratio: 1.2:1). The age of the patients ranged from 30 to 70 years, with an average age of 49.3 years. Four patients were firstly diagnosed, while seven patients had recurrent tumors. All patients received preoperative imaging examinations including enhanced CT, MRI, and other auxiliary examinations of the parotid gland. The surgical procedures were performed at the Department of Oral and Maxillofacial Surgery, School and Hospital of Stomatology, Wuhan University, and the postoperative pathological analyses were conducted in the Department of Oral Pathology, School and Hospital of Stomatology, Wuhan University. The cases comprised 2 SC, 2 SCC, 2 carcinosarcoma, 1 MEC, 1 EMC, 1 SDC, 1 BCC, and 1 osteosarcoma.

**Table 1:** General information of the 11 cases who experienced soft tissue flap reconstruction for postoperative defects following resection of malignant tumors in parotid region

NO.	Gender	Age (year)	First diagnosis/Recurrence	Nerve function (Post)	Nerve function(Pre)	Cervical Lymphatic dissection	Lymph node metastasis	Flap type	Pathology	Postoperative condition	Follow-up
1	Female	30	First diagnosis	Well	Well	Yes	No	Anterolatera thigh flap	SC	Recurrence(2020–04;2020–09);^125^I seed implantation(2020–11)	Alive
5	Male	30	First diagnosis	Incomplete eye closure on the right side	Well	Yes	Yes	Anterolateral thigh flap	SCC	Radiotherapy	Alive
11	Male	43	Recurrence	–	Well	Yes	No	Anterolateral thigh flap	Osteosarcoma	Radiotherapy	Alive
6	Male	47	Recurrence	–	Incomplete eye closure	Yes	No	Pectoralis major muscle flap	SDC	Radiotherapy	Alive
9	Female	47	Recurrence	–	Corner of the mouth slanting, incomplete eye closure on the right side	No	No	Anterolateral thigh flap	SC	None	Alive
2	Female	52	Recurrence	–	Corner of the mouth slanting	Yes	No	Anterolateral thigh flap	Carcinosarcoma	Radiotherapy	Death (hypertension)
3	Female	54	First diagnosis	Well	Well	No	No	Anterolateral thigh flap	MEC	None	Alive
4	Male	54	First diagnosis	Incomplete eye closure on the right side	Well	Yes	No	Anterolateral thigh flap	SCC	Radiotherapy	Alive, lung metastases, immunotherapy
10	Female	56	Recurrence	–	Incomplete eye closure on the right side, corner of the mouth slanting	Yes	No	Forearm flap	BCC	2014:Recurrence	Lost to follow-up
7	Male	59	Recurrence	–	Well	Yes	No	Pectoralis major muscle flap	Carcinosarcoma	–	Lost to follow-up
8	Male	70	Recurrence	–	Incomplete eye closure on the right side	No	No	Anterolateral thigh flap	EMC	None	Alive

**Table 2:** Statistical analysis of the 11 cases

Variable	Number (%)
**Age**	
21–30	2 (18.2%)
31–40	0
41–50	3 (27.2%)
51–60	5 (45.5%)
61–70	1 (9.1%)
**Gender**	
Male	6 (54.55%)
Female	5 (45.45%)
**First diagnosis /recurrence**	
First diagnosis	4 (36.36%)
Recurrence	7 (63.64%)
**Cervical Lymphatic dissection**	
Yes	8 (72.73%)
No	3 (27.27%)
**Lymph node metastasis**	
Yes	1 (12.5%)
No	7 (87.5%)
**Type of flaps**	
Anterolateral thigh flap	8 (72.73%)
Forearm flap	1 (9.09%)
Pectoralis major muscle flap	2 (18.18%)

### Treatment of the patients and outcomes

**Fig. 2:**
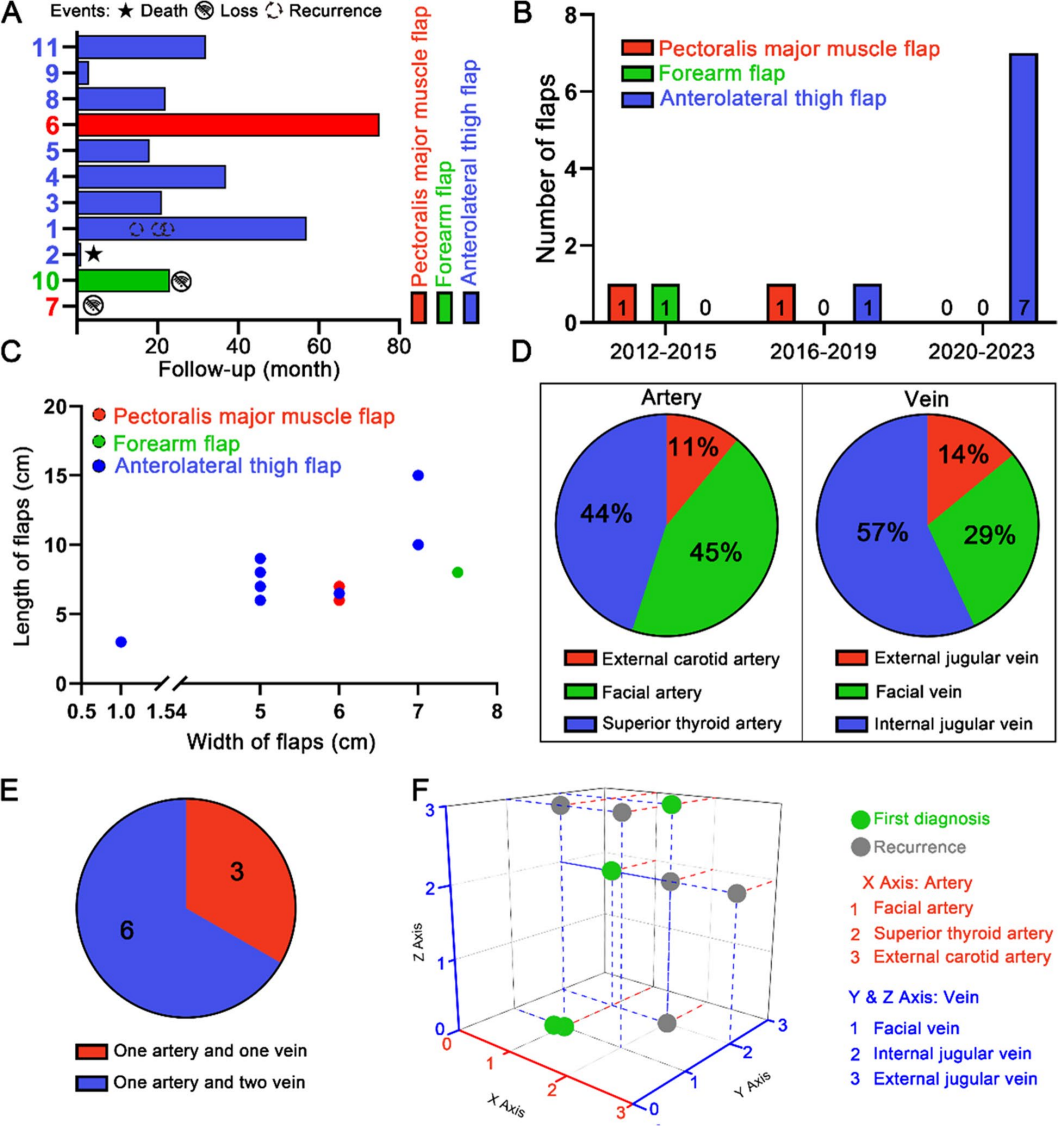
Treatment, outcomes and of soft tissue flaps’ characteristics in the reconstruction in parotid region. **A** The postoperative outcomes of patients with parotid malignant tumors who underwent soft tissue flap reconstruction for defects. **B** Comparative analysis of the annual trends in the utilization of soft tissue flaps in the parotid region. **C** Size variations of soft tissue flaps used for postoperative defect reconstruction following resection of malignant tumors in the parotid region. **D** Distribution of different arteries and veins used for soft tissue free flaps in the parotid region. **E** Count of soft tissue flaps consisting of 1 artery and 2 veins, or 1 artery and 1 vein. **F** Number of arteries and veins in each free flap, along with specific nomenclature of the blood vessels

In this group of 11 cases, comprehensive treatment primarily involving surgery was implemented. Eight patients underwent resection of parotid malignant tumors along with ipsilateral neck lymph node dissection. Among the 8 cases, 3 cases were initial diagnoses, including 2 cases of SCC, and 1 case of SC. Additionally, 5 cases were recurrent tumors, encompassing 2 cases of carcinosarcoma, 1 case of SDC, 1 case of osteosarcoma, and 1 case of BCC. Among the 8 cases, one case of SCC showed lymph node metastasis upon pathological examination. Regarding the cases with initial diagnosis, 2 cases received postoperative radiotherapy, and 1 case underwent ^125^I seed implantation therapeutic treatment after experiencing two recurrences. The follow-up period for this group of patients concluded on June 30, 2023 (Fig. 2A). There were 2 instances of local tumor recurrence that necessitated additional surgery (1 case of SC and 1 case of BCC). Two patients were lost to follow-up (1 case of carcinosarcoma and 1 case of BCC). The post-treatment survival outcomes are as follows: 1 case exhibited distant metastasis (SCC of the parotid gland metastasized to the lungs), and 1 case resulted in death due to a non-cancer related disease (hypertension).

### The characteristics of soft tissue flaps in the reconstruction in parotid region

Soft tissue flap reconstruction methods were utilized to address post-tumor resection defects, which consisted of 8 cases with anterolateral thigh flaps, 2 cases with pectoralis major muscle flaps, and 1 case with a forearm flap. Interestingly, we found that among the surgical patients from 2012 to 2015, only one received repair using a pectoralis major muscle flap, and the other underwent repair with a forearm flap. For the surgical patients between 2016 and 2019, also only one underwent repair with a pectoralis major muscle flap, the other received repair using an anterolateral thigh flap. However, from 2020 to 2023, all seven patients underwent repair and reconstruction using an anterolateral thigh flap (Fig. 2B). The flap sizes ranged from approximately 1 cm × 3 cm to 7 cm × 15 cm (Fig. 2C). The recipient vessels for flap anastomosis included 4 cases with the facial artery, 4 cases with the superior thyroid artery, and 1 case with the external carotid artery. The recipient vein anastomosis ratio was 57% for branches of the internal jugular vein, 29% for the facial vein, and 14% for the external jugular vein (Fig. 2D). Specifically, for each soft tissue free flap, there are a total of 6 flaps, consisting of 1 artery and 2 veins. Among the remaining 3 flaps, there is 1 artery and 1 vein (Fig. 2E). Figure 2F shows the number of arteries and veins for each free flap, as well as the specific nomenclature of the blood vessels.

### The characteristics of anterolateral thigh flap in the reconstruction in parotid region

**Fig. 3:**
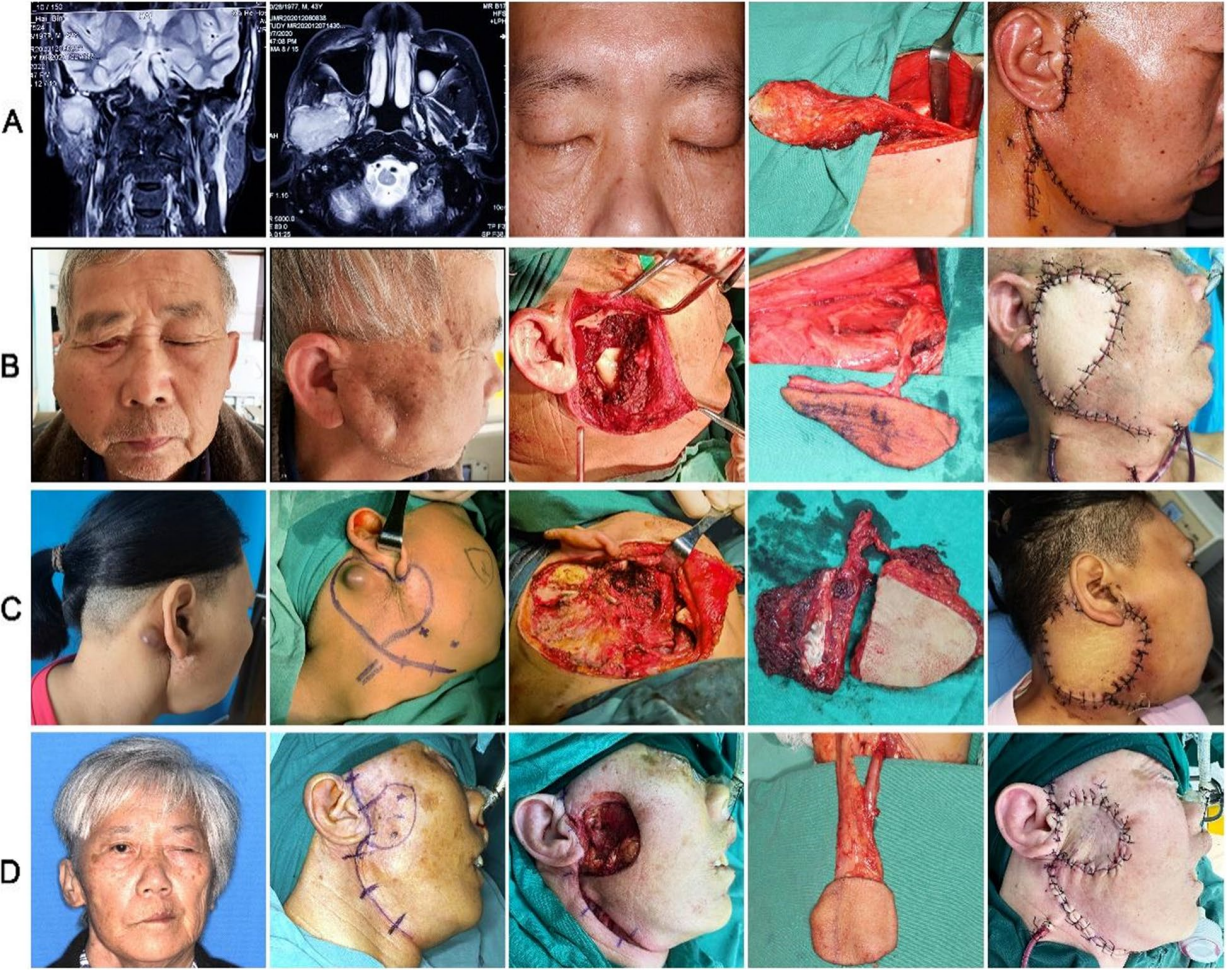
Images of representative cases using anterolateral thigh flaps. **A** A 43-year-old male patient with radiation-induced sarcoma (RIS) underwent repair of a deep tissue defect using a gracilis muscle flap harvested from the anterior-lateral thigh. **B** A 70-year-old male patient diagnosed with right parotid gland EMC received repair for a skin tissue defect utilizing an anterior-lateral thigh skin flap. **C** A 47-year-old female patient with right parotid gland SC underwent repair for both skin and deep tissue defects using a anterolateral thigh chimeric perforator myocutaneous flap. **D** A 56-year-old female patient with right parotid gland BCC underwent repair for both skin and subcutaneous tissue defects using a forearm flap

**Fig. 4:**
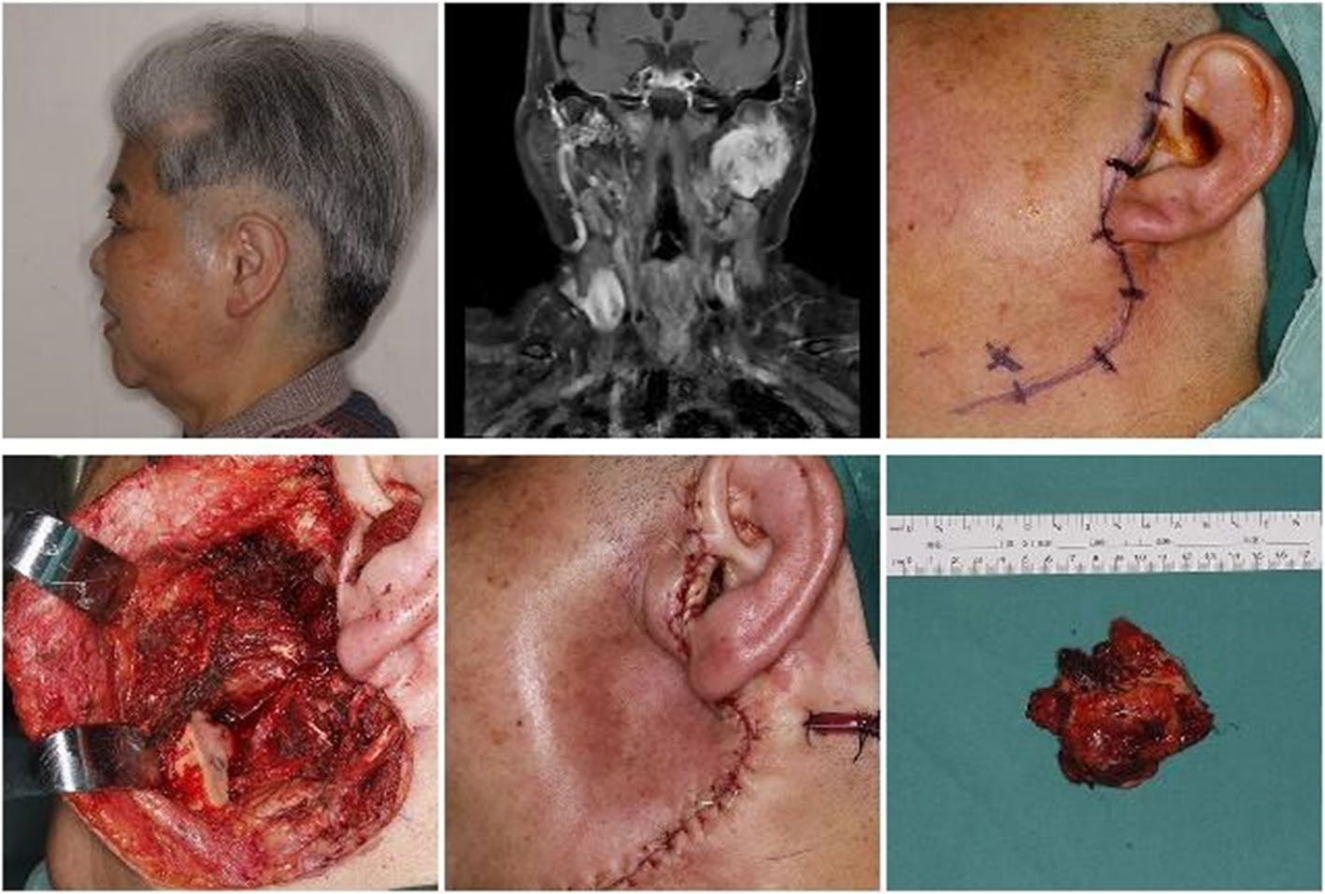
A 76-year-old female patient with left parotid gland ACC without using flap for subcutaneous tissue defects

Since the anterolateral thigh flap is the most commonly used flap in these 11 patients, we proceeded with a comprehensive analysis of their specific characteristics. Anterolateral thigh muscle flap was used to fill the substantial deep defect which did not involve a skin defect in 2 patients (Fig. 3A). Three patients with skin defects underwent reconstruction using the anterolateral thigh flap (Fig. 3B). The anterolateral thigh chimeric perforator myocutaneous flap, which provides unique advantages for combined defects involving both skin and muscle, was employed in 3 patients (Fig. 3C). In comparison, the forearm flap was capable of reconstructing a limited range of defects in the parotid region (Fig. 3D). Without flap was used to fill the defect, the skin would appear visibly depressed (Fig. 4).

## Discussion

### Selection of treatment plan for malignant tumors in the parotid region

In regards to SC, the salivary gland is considered the primary site [8]. In this article, one patient was identified as a primary case and underwent removal of the parotid gland and neck lymph nodes. Immediate reconstruction was performed using the anterolateral thigh flap. No lymph node metastasis occurred, and adjuvant therapies such as chemotherapy or radiation were not administered post-surgery. However, recurrence was observed after 2 years, leading to resection and the implantation of radioactive ^125^I seeds. In another case, the tumor in the parotid region was removed without neck lymph node removal, followed by the implantation of radioactive ^125^I seeds. Recurrence was observed after 4 years, and the patient underwent tumor excision with immediate anterolateral thigh flap reconstruction. No neck lymph node dissection was performed, and no distant metastasis was detected. According to relevant literature reports, SC have a low rate of lymph node metastasis (6.1%) and a distant metastasis rate of only 3.4%. Therefore, routine neck lymph node dissection may not be necessary [9]. In this article, one case of MEC was identified as a primary case with no lymph node metastasis observed in the neck. For this patient, only lesion excision and simultaneous reconstruction using the anterolateral thigh flap were performed. Pathological results indicated moderate malignancy, and no adjuvant radiation and chemotherapy were administered post-surgery. Our viewpoint is that simple lesion excision can be considered for clinical stage I and II cases with high differentiation and less obvious symptoms. Neck lymph node dissection is generally not recommended unless there are clear signs of enlarged lymph nodes suggestive of metastasis. Spiro et al. [10]. reported that the rate of neck lymph node metastasis is approximately 10% for pathological grades I to II and stages I to II. For pathological grade III and stages III to IV, the rate increases significantly to as high as 44%. These findings further support our viewpoint.

SCC originating from the parotid gland is an extremely rare occurrence, accounting for only 0.3–3.4% of malignant tumors in the salivary gland, with an average incidence of 2.1% [11, 12]. In this article, 2 cases with SCC are primary originating from the salivary gland region. They underwent extensive resection of the parotid region lesion and neck lymph node dissection, followed by adjuvant radiotherapy. In one case, metastasis to the cervical IIB lymph nodes was observed; in the other case, it subsequently developed into lung metastasis after 1 year. The latter patient is currently undergoing immunotherapy. Based on these observations, for primary SCC in the parotid region, we recommend a comprehensive treatment approach that includes combined radical surgery (ipsilateral neck lymph node dissection of I-III) and adjuvant radiation and chemotherapy.

Both carcinosarcoma [13] and SDC [14] are discussed as rare and highly malignant tumors in the parotid region. These tumors exhibit high invasiveness and have a poor prognosis. Therefore, the current treatment primarily relies on surgery, and the effectiveness of postoperative radiation and chemotherapy requires further research. On the other hand, EMC [15] is characterized by a lower degree of malignancy, and surgery is the preferred treatment option. The rate of lymph node metastasis is relatively low, but selective neck lymph node dissection may be considered if the tumor is extensively involved. The case of osteosarcoma presented in this article is a rare complication known as radiation-induced sarcoma (RIS) [16, 17], which is an uncommon consequence of radiation therapy. The patient had a history of MEC in the parotid gland, along with a surgical and radiation history. The mechanism of RIS is not fully understood, but surgical treatment is the primary approach. The BCC cases involve malignancies in the parotid region [18], and the treatment method is surgical resection followed by postoperative radiation. Radical tumor resection can be an important means of symptom control.

### Selection of soft tissue flaps for the reconstruction for postoperative defects following resection of malignant tumors

Advanced malignant tumors of the parotid gland often lead to complex tissue defects after surgical removal, which can include facial skin, chewing muscles, and the mandible. Prompt repair and reconstruction are necessary in such cases [19]. The utilization of pedicled pectoralis major muscle flaps, and free flaps including anterolateral thigh flaps and forearm flaps has demonstrated significant advantages in restoring facial aesthetics. The pectoralis major muscle flap, being a conventional tissue flap, is widely employed and carries a pedicle, thus minimizing the risk of vascular complications [20]. In this study, both patients underwent radical neck dissection, and the utilization of the pectoralis major muscle flap to cover the carotid artery resulted in successful contour reconstruction after the procedure.

The anterolateral thigh flap is highly versatile and widely used, often referred to as the “universal flap”. This flap possesses a long vascular pedicle, matching vessel diameter with neck vessels, multiple accompanying arteries and veins to ensure smooth blood flow, and the ability to harvest a sizable flap area [21–23]. Our experience suggests selecting the appropriate type of anterolateral thigh tissue flap based on the extent of the defect during surgery.We categorized the defects in this region into three types. Type I: The skin in the surgical area remains intact but there exists a substantial deep defect, the anterolateral thigh muscle flap can be utilized to fill the cavity, eliminate dead space, and reduce facial concave deformities. Type II: There is only a soft tissue defect without involvement of the mandible or zygomatic arch, immediate coverage with an anterolateral thigh flap can prevent skin tension and poor wound healing resulting from nearby flap recruitment and closure. Type III: Tumors involving the mandible, zygomatic arch, facial nerve, and external ear often result in extensive soft tissue defects with deep and varied cavities. In such cases, the anterolateral thigh chimeric perforator myocutaneous flap offers unique advantages. Unlike traditional composite flaps, this flap and muscle flap share a common vascular pedicle but are not rigidly fixed together, permitting independent and flexible positioning adjustments to repair different areas. It provides enhanced safety and highly satisfactory outcomes.

Our data demonstrates an increased frequency of anterolateral thigh flap usage for parotid region defect reconstruction in recent years at our hospital compared to other types. This could be attributed to the advancements in microsurgery and the maturation of vascular anastomosis techniques, which have provided greater flexibility and advantages to free flaps over pedicled pectoralis major muscle flaps. Additionally, forearm flaps may not offer sufficient soft tissue volume so that narrow indications of the reconstruction requirements for this region [24]. Therefore, for large defects in the parotid region, we highly recommend the use of anterolateral thigh muscle flaps, skin flaps, or chimeric perforator myocutaneous flaps for reconstruction.

In recent years, some researchers have achieved favorable outcomes using free peroneal artery chimeric perforator flaps to repair postoperative defects resulting from extensive local lesions of advanced parotid gland cancer. Its advantage lies in simultaneous repair of bone defects. However, drawbacks such as prolonged bed rest and uneven residual skin tissue in the lower leg surgical area exist, and this technique has not yet gained widespread adoption [25].

### Selection of recipient vessels for free flaps

The selection of suitable recipient vessels plays a crucial role in the success of free flap transplantation [26]. Based on the statistical data from this study, the most commonly utilized recipient arteries include the facial artery, superior thyroid artery, and others. Likewise, the commonly used recipient veins comprise the facial vein, external jugular vein, internal jugular vein, and their respective branches. For patients with malignant tumors of the parotid gland who have not undergone neck lymph node dissection, the facial artery, facial vein, and external jugular vein can be chosen as recipient vessels. However, for patients with a history of previous neck surgery and/or radiotherapy, as well as recurrent or refractory cases, microsurgical repair and reconstruction can be challenging due to limited vessel availability, severe fibrosis, and complex tissue anatomy [27]. In such scenarios, the superior thyroid artery, external carotid artery, and branches of the internal jugular vein can be utilized as recipient vessels. In some instances, the transverse cervical artery and superficial temporal artery have been suggested by researchers due to consistent anatomy and their location usually outside the radiation field. These vessels offer viable options for micro-reconstruction of recipient vessels in cases with vascular deficiencies in the neck region [28–30]. Considering the complexity of vascular conditions in the donor sites of these patients, especially those with recurrence or a history of radiotherapy, our experience suggests the recommendation of selecting one artery and two veins for anastomosis whenever possible. This approach ensures a smooth blood supply and enhances the survival rate of the flaps.

## Conclusion

Malignant tumors of the parotid region, particularly advanced and recurrent tumors, often necessitate immediate soft tissue flap reconstruction following extensive excision surgery. This approach serves to restore facial appearance, align with surgical aesthetics, and effectively meet the patient’s expectations for surgical outcomes. A comprehensive treatment approach, primarily focusing on surgery, is commonly utilized based on preoperative imaging examination, intraoperative assessment, and postoperative pathological diagnosis. The selection and design of flap types and sizes should be determined based on the extent of the defect. In choosing recipient vessels for anastomosis, factors such as the patient’s history of neck lymph node dissection and previous radiotherapy should be taken into consideration. By considering these factors, the overall treatment outcome for this disease can be enhanced.

## Data Availability

All data generated or analysed during this study are included in this published article.
